# Laser Processing of Polymer Films Fabricated from PHAs Differing in Their Monomer Composition

**DOI:** 10.3390/polym13101553

**Published:** 2021-05-12

**Authors:** Tatiana G. Volova, Alexey I. Golubev, Ivan V. Nemtsev, Anna V. Lukyanenko, Alexey E. Dudaev, Ekaterina I. Shishatskaya

**Affiliations:** 1 Basic Department of Biotechnology, School of Fundamental Biology and Biotechnology, Siberian Federal University, 79 Svobodnyi Av., 660041 Krasnoyarsk, Russia; ivan_nemtsev@mail.ru (I.V.N.); lav@iph.krasn.ru (A.V.L.); alex15-96@mail.ru (A.E.D.); shishatskaya@inbox.ru (E.I.S.); 2 Institute of Biophysics SB RAS, Federal Research Center “Krasnoyarsk Science Center SB RAS”, 50/50 Akademgorodok, 660036 Krasnoyarsk, Russia; 3 L.V. Kirensky Institute of Physics SB RAS, Federal Research Center “Krasnoyarsk Science Center SB RAS”, 50/38 Akademgorodok, 660036 Krasnoyarsk, Russia; golubev@ksc.krasn.ru; 4 Special Design and Technological Bureau ‘Nauka’ Federal Research Center “Krasnoyarsk Science Center SB RAS”, 50/45 Akademgorodok, 660036 Krasnoyarsk, Russia; 5 Federal Research Center “Krasnoyarsk Science Center of the Siberian Branch of the Russian Academy of Sciences” 50 Akademgorodok, 660036 Krasnoyarsk, Russia

**Keywords:** P(3HB), copolymers, films, CO_2_ laser, SEM, AFM, water contact angles, MTT assay, NIH 3T3 fibroblasts

## Abstract

The study reports results of using a CO_2_-laser in continuous wave (3 W; 2 m/s) and quasi-pulsed (13.5 W; 1 m/s) modes to treat films prepared by solvent casting technique from four types of polyhydroxyalkanoates (PHAs), namely poly-3-hydroxybutyrate and three copolymers of 3-hydroxybutyrate: with 4-hydroxybutyrate, 3-hydroxyvalerate, and 3-hydroxyhexanoate (each second monomer constituting about 30 mol.%). The PHAs differed in their thermal and molecular weight properties and degree of crystallinity. Pristine films differed in porosity, hydrophilicity, and roughness parameters. The two modes of laser treatment altered these parameters and biocompatibility in diverse ways. Films of P(3HB) had water contact angle and surface energy of 92° and 30.8 mN/m, respectively, and average roughness of 144 nm. The water contact angle of copolymer films decreased to 80–56° and surface energy and roughness increased to 41–57 mN/m and 172–290 nm, respectively. Treatment in either mode resulted in different modifications of the films, depending on their composition and irradiation mode. Laser-treated P(3HB) films exhibited a decrease in water contact angle, which was more considerable after the treatment in the quasi-pulsed mode. Roughness parameters were changed by the treatment in both modes. Continuous wave line-by-line irradiation caused formation of sintered grooves on the film surface, which exhibited some change in water contact angle (76–80°) and reduced roughness parameters (to 40–45 mN/m) for most films. Treatment in the quasi-pulsed raster mode resulted in the formation of pits with no pronounced sintered regions on the film surface, a more considerably decreased water contact angle (to 67–76°), and increased roughness of most specimens. Colorimetric assay for assessing cell metabolic activity (MTT) in NIH 3T3 mouse fibroblast culture showed that the number of fibroblasts on the films treated in the continuous wave mode was somewhat lower; treatment in quasi-pulsed radiation mode caused an increase in the number of viable cells by a factor of 1.26 to 1.76, depending on PHA composition. This is an important result, offering an opportunity of targeted surface modification of PHA products aimed at preventing or facilitating cell attachment.

## 1. Introduction

The development of new, environmentally friendly materials, which will completely degrade without releasing toxic products, joining the global cycle, is the priority for critical technologists of the 21st century. Annual production of non-degradable synthetic plastics has exceeded 380 million tons, and it continues increasing [1,2,3,4]. In developed countries, no more than 16–18% of plastic waste is recycled [5]. It is mostly landfilled, posing a global environmental problem [6,7], causing large-scale pollution, upsetting the stability and disrupting the structure of natural ecosystems, and threatening human health [8]. That is why it is important to research and develop biodegradable plastics as an alternative to synthetic materials [9,10].

Polyhydroxyalkanoates (PHAs), degradable polyesters of microbial origin, are promising “green” plastics, which are degraded by natural microflora to CO_2_ and H_2_O, causing no harm to biota and the entire environment [11,12,13,14,15,16,17]. PHAs are synthesized by prokaryotes from various substrates, including waste products [18,19,20,21]. Development of PHAs was a notable event for biotechnology of degradable materials [6,22,23,24,25,26,27,28]. Monomer composition of PHAs determine their basic properties (crystallinity, thermal and molecular-weight properties, biodegradability), which vary widely, enabling fabrication of products with diverse physical/mechanical characteristics [15,29,30,31,32,33]. Being UV resistant, non-hydrolyzed in liquid media, and thermoplastic, PHAs are processable from different phase states (solution, emulsion, powder, melt) by available techniques [34,35,36]. These useful properties, along with biodegradability and high biological compatibility, make PHAs promising materials of the 21st century and serious competitors of the common biodegradable plastics (polylactide, polyethylene terephthalate, polyamides, etc.) in various applications—from municipal engineering and agriculture to pharmacology and biomedicine. PHAs hold the greatest promise for developing biomedical products and devices, including nonwoven and disposable products, sutures and wound dressings, controlled drug delivery systems, scaffolds for cell and tissue engineering, components for reconstructive surgery and transplantation [14,37,38,39].

The most common and the best studied PHA, which is manufactured on the largest scale, is homopolymer of 3-hydroxybutyric acid (poly-3-hydroxybutyrate, P(3HB)). Despite the great potential of this polymer, its high crystallinity (above 70%) and hydrophobicity limit its use. P(3HB) does not crystallize to form an ordered structure, it is difficult to process P(3HB) into products, which demonstrate low shock resistance and rigidity and are prone to “physical ageing” [40,41]. P(3HB)-based products are degraded slowly, and implants may cause pronounced foreign body reaction [42,43]. Properties of polymeric materials, including P(3HB), can be improved by using biological, chemical, and physical methods, such as fabrication of P(3HB) composites with other materials, biosynthesis of PHA copolymers, chemical modification, and physical treatment of the surface of polymer products [6,25,27,44,45,46,47,48]. These methods help, more effectively or less effectively, change the properties of polymer products, increase their biodegradation rate, enhance their flexibility and mechanical strength, increase surface hydrophilicity and porosity to facilitate cell attachment, improve gas dynamic properties of the products, and enhance their permeability to substrates and metabolic products of cells and tissues.

Laser treatment is a relatively new approach to modification of polymer products. Its main advantage over other treatments is that it modifies the surface selectively, without destroying the material or producing toxic substances. The studies published so far address the influence of various types of laser treatment (using CO_2_, picosecond, excimer lasers) on the surface of various polymers: polycaprolactone, [49,50] polymethylsiloxane [51], polyethylene terephthalate [52,53], poly-L-lactic-acid [54,55], poly(L-lactide-co-glycolide) [56], chitosan, and chitosan/ceramics composites [57].

A review by Ravi-Kumar et al. [58] demonstrated for a number of polymers (PMMA, PET, and PTFE) that the effect of laser treatment was determined by the type and composition of polymer material, laser parameters, and treatment mode. These data and analysis of very many available studies suggest that smaller pulse duration results in more considerable vaporization of material. A higher melting temperature can cause formation of a large heat affected zone. Cracks and non-uniformity due to melt expulsion from the heating zone and higher molecular weight of the polymer can decrease ablation rate because of the formation of highly viscous molten material, and larger thermal conductivity can result in a larger heat affected zone, especially with long pulse duration.

One of the new processes of adding texture or picture onto the effective surface of polymer products is laser surface texturing (LST) [59]. A number of studies showed the high potential of LST for modifying and enhancing the effectiveness of biomedical materials. For instance, laser treatment at different wavelengths (532 nm and 355 nm) of the surface of carbon-coated polyethylene film increased surface wettability and roughness and favorably influenced cell—material interaction [60]. CO_2_-laser texturing of thin poly(L-lactide) (PLLA) films improved surface microhardness, roughness, wettability, and cytocompatibility. The authors of that study demonstrated that CO_2_-laser texturing of PLLA films adjusted physical and structural surface properties of the material and considerably changed its mechanical properties [61].

Shivakoti et al. [59] presented an in-depth review of laser surface texturing for biomedical applications. The researchers used various types of lasers, such as CO_2_ laser, excimer laser, fiber laser, etc., to produce texture to explore the efficacy of the process and its impact on proliferation, osseointegration, cell adhesion, etc. and adapt structural and physical properties of film surface to cell engineering applications. Daskalova et al. [62] reported using femtosecond laser modification to produce various structures of thin films of biodegradable polymers and their ceramic blends (pure chitosan thin films and different percentage of composite blends of chitosan (Ch)/HAp/ZrO_2_ thin films). Laser treatment increased film surface roughness and enhanced attachment and orientation of eukaryotic cells. A similar favorable effect of CO_2_-laser treatment of thin poly(L-lactide) (PLLA) films was described in another study [61], which showed the effect of laser treatment on film surface microhardness, roughness, wettability, and cytocompatibility. The authors concluded that CO_2_-laser texturing of PLLA films was able to adjust physical and structural surface properties of the material in spite of the considerable change in its mechanical properties.

Analysis of the most recent published studies, including a thorough review by Professor Ravi-Kumar et al. [58], key experimental works [28,48,54,55], and the latest studies [56,57,58,59,61,62] has provided the basis for summarizing the currently available data and existing notions of the effects produced by laser treatment of polymer materials. Laser ablation is widely used today to treat various materials (metals, ceramics, glass, and polymers). Polymers attract considerable interest because of their unique properties such as light weight, corrosion resistance, lower friction properties and less wear compared to metals, and high application potential especially for biomedicine. Laser ablation is the top-down process of removing material by focusing the laser beam onto the material. Ablation occurs only when the material absorbs enough energy to be melted or vaporized. “Laser ablation” is interpreted variously in different works. The reason is that the processes occurring when laser irradiation affects the polymer are very complex: thermal, thermo-oxidative, and/or mechanical breakdown processes take place simultaneously, resulting in vaporization of fragments of macromolecules (even oligomers), and sometimes, separation of polymer and filler particles by a gas or plasma plume [58].

Laser ablation has become a powerful method of creating micro- and nano-structures on surfaces of products fabricated from various polymers. The general mechanism of laser ablation is the same in all laser treatments such as laser beam drilling, high-precision drilling, and laser cutting. Ablation is a combination of vaporization and/or melt expulsion. When a focused beam of laser radiation hits the surface, electrons present in the material are excited by laser photons, which results in generation of heat by absorbing photon energy. This is consistent with Beer–Lambert law, stating that the amount of light absorbed is dependent on the thickness of the material and light source intensity. The transition from solid to gas causes the formation of a plasma plume. This phase transition consists of several steps. First, the heat produced by absorption of laser photons causes the formation of a melt pool. The temperature increases because of the incoming pulses, the melt reaches the vaporization state, and high pressure is created, which ejects molten material from the pool. As the temperature rises further in the zone of laser—material interaction, the liquid reaches the liquid–vapor phase transition stage. This mechanism is usually observed during ablation using long-pulsed lasers and can be referred to as a “burst”. With ultrashort laser pulses, absorption becomes nonlinear and dependent on intensity. The bound electrons of the material can be directly ionized by large absorption coefficient and due to high light intensity. Various mechanisms of laser treatment effects are dependent on the combination of specific properties of light and material. Laser ablation of polymers depends on a great number of factors such as laser wavelength. Depending on the properties of the laser and the material, such as fluence, absorption coefficient, reflectance, wavelength, and pulse duration, the ablation mechanism can be purely chemical, thermal, or a combination of both. Photochemical ablation occurs because of the breaking of covalent bonds in polymer chains due to the energy of the UV photons. Photothermal ablation considers the electron excitation by the UV photons to be thermalized, which results in the breaking of polymer bonds.

The most powerful and commonly used continuous wave lasers are CO_2_ gas discharge lasers. In these lasers, light amplification occurs due to carbon dioxide molecules. Radiation is mainly generated at a wavelength of 10.6 µm. The efficiency of such lasers is higher than 10%, and they can generate high-quality radiation powers of several kilowatts. CO_2_ lasers are commonly used for processing different materials, e.g., cutting, welding, and engraving, and in laser surgery. It has been assumed that treatment with laser radiation makes the surface more hydrophilic, creating regions facilitating the attachment of cultivated cells [63].

Laser treatment of the surfaces of PHA-based products has been addressed in rather few studies, but research done using certain PHAs, mainly homogeneous P(3HB), showed that laser treatment could alter the surface and bulk properties of P(3HB)-based products [64,65,66,67,68].

As noted above, PHAs comprise polymers with different chemical composition [69]. However, very few publications can be found that discuss laser treatment of PHA copolymers and modification of their properties. Ortiz et al. [28] reported picosecond laser ablation of films of P(3HB) and medium-chain-length poly(3-hydroxy octanoate-co-3-hydroxy decanoate) and analyzed changes in the film surface topography. Other authors [70,71,72] described using Nd:YAG laser and KrF excimer laser to treat films of poly(3-hydroxybutyrate-co-3-hydroxyvalerate) prepared by solving casting method and showed modification of surface topography including pore formation and changes in roughness.

The present study was the first to investigate the effect of laser treatment on the surface microstructure and properties of polymer films of four PHA types—poly-3-hydroxybutyrate and three copolymers of 3-hydroxybutyrate: with 4-hydroxybutyrate, 3-hydroxyvalerate, and 3-hydroxyhexanoate.

## 2. Materials and Methods

### 2.1. Materials

PHAs were synthesized using the Cupriavidus necator B-10646 bacterial strain and proprietary technology [73]. The strain is registered in the Russian National Collection of Industrial Microorganisms (VKPM). A two-stage process was used. In the first stage (30–35 h), cells were grown in the mineral salt medium under limited urea (nitrogen source) supply (the amount of nitrogen supplied in this stage was 50% of the cells’ physiological requirements—0.5 g nitrogen/g biomass). In the second stage (30–35 h), cells were cultured in nitrogen-free medium. The main carbon substrate was glucose (ZAO Khimreaktivsnab, Ufa, Russia) at a concentration of 10 g/L. To synthesize PHA copolymers, the cell culture was supplemented with precursors of the target monomers: salts of valeric and hexanoic acids and γ-butyrolactone (Sigma-Aldrich, Saint Louis, MO, USA) at a concentration of 1.0–1.5 g/L. The doses of the precursors were measured to obtain similar fractions of the second monomers in the different types of copolymers. The dosed feeding of precursor substrates to the bacterial culture in the mode of PHA synthesis resulted in the synthesis of polymers with different composition: homopolymer of 3-hydroxybutyrate P(3HB) [−O−CH(CH3)−CH2−CO−] and copolymers, each consisting of the 3-hydroxybutyrate monomer and another monomer. Second monomers differed in their structure and carbon chain length: 4-hydroxybutyrate (4HB) [−O−CH2−CH2−CH2−CO−], 3-hydroxyvalerate (3HV) [−CH(C2H5)−CH2−CO−], and 3-hydroxyhexanoate (3HHx) [−O−CH(C3H7)−CH2−CO−].

### 2.2. PHA Recovery from Cell Biomass

Polymer was extracted from cell biomass with dichloromethane, concentrated using an R/210V rotary evaporator (Büchi, Flawil, Switzerland), and precipitated with ethanol. Polymer was re-dissolved and re-precipitated to remove impurities and prepare homogeneous specimens. Chemical purity of the specimens was detected using a 7890A gas chromatograph equipped with a 5975C chromatograph-mass spectrometer (Agilent Technologies, Santa Clara, CA, USA). The polymer was dried in the laboratory vent hood at room temperature for 72 h.

### 2.3. PHA Chemical Composition

Purity of the polymer and copolymers was determined by chromatography of methyl esters of fatty acids after methanolysis of purified polymer samples using a 7890A chromatograph-mass spectrometer (Agilent Technologies, Santa Clara, CA, USA) equipped with a 5975C mass detector (Agilent Technologies, Santa Clara, CA, USA) [74].

### 2.4. Physicochemical Properties of PHAs

Physicochemical properties of PHAs were examined using high performance liquid chromatography, X-ray structure analysis, and differential scanning calorimetry. All methods are described in detail elsewhere [75]. Physicochemical properties of the specimens are provided in Table 1.

The weight average molecular weight (M_w_) and polydispersity (Ð) of PHAs were examined with a gel permeation chromatograph (Agilent Technologies 1260 Infinity, Waldbronn, Germany). Thermal analysis of PHA specimens was performed using a DSC-1 differential scanning calorimeter (METTLER TOLEDO, Schwerzenbach, Switzerland). Melting point (T_melt_) and thermal degradation temperature (T_degr_) were determined from endothermic peaks in thermograms. X-Ray experiments were performed to determine crystallinity of PHA specimens employing a D8 ADVANCE diffractometer (Bruker, AXS, Karlsruhe, Germany) equipped with a VANTEC fast linear detector. The degree of crystallinity (C_x_) was calculated as a ratio of the total area of crystalline peaks to the total area of the radiogram (the crystalline + amorphous components). The measurement error was 3%.

### 2.5. Production of Polymer Films

Films were prepared by casting a 2% polymer solution in dichloromethane in degreased Teflon-coated molds, and then the films were left to stay in a laminar flow cabinet (Labconco, Kansas City, MO, USA) for 72 h. Afterwards, they were dried in a vacuum desiccator (Labconco, Kansas City, MO, USA) or a thermostatically controlled cabinet at 40 °C (dichloromethane evaporation-boiling point) until the solvent completely evaporated. Discs of diameter 10 mm were cut out using a template. The membranes were sterilized using H_2_O_2_ plasma in a Sterrad NX sterilization system (Johnson & Johnson, Irvine, CA, USA).

### 2.6. Laser Treatment Modes

Laser treatment of the surface of flexible transparent polymer films was performed by moderate uniform irradiation of the surface, using CO_2_ laser LaserPro Explorer II (Coherent, Santa Clara, CA, USA). CO_2_ laser LaserPro Explorer II has the following characteristics: wavelength 10.6 µm, maximum power 30 W, and maximum speed 2 m/s in the modes of raster and vector engraving, at a maximum resolution of 1000 dpi. The laser is equipped with a standard SeZn lens, F = 2’. The varied parameters were power and speed of processing and processing modes: in the focused mode, continuous wave treatment was performed linearly (line by line) and in the quasi-pulsed mode, raster engraving (point by point) was used. Power was 3 and 13.5 W; speed was 1 and 2 m/s.

### 2.7. A Study of Polymer Film Surface

Methods of studying film surface are described in detail elsewhere [75].

The thickness of the films was measured with a 25–0.001 electronic digital micrometer (Schut Geometrical Metrology, Groningen, Netherlands). Porosity of the films was determined from SEM images using a software package for digital image analysis (free open-source software package for scientific analysis, editing, and processing of raster images), Image J v1.52.

The surface microstructure of PHA films was analyzed using scanning electron microscopy (FE-SEM S 5500 high-resolution scanning electron microscope Hitachi, Tokyo, Japan). Prior to microscopy, the samples were sputter coated with platinum (at 25 mA, for 60 s), using an EM ACE200 (Leica, Vienna, Austria). Surface properties were studied with a Drop Shape Analyzer—DSA-25E (Krüss, Hamburg, Germany) using the DSA-4 software for Windows. The Owens, Wendt, Rabel, and Kaelble method was used to calculate surface free energy and its dispersion and polar components (mN/m).

The roughness of film surface was determined using atomic-force microscopy (AFM) in semicontact mode (DPN 5000, NanoInk, Skokie, IL USA). The arithmetic mean surface roughness (Sa) and the root mean square roughness (Sq) were determined based on 10 points, as the arithmetic averages of the absolute values of the vertical deviations of the five highest peaks and lowest valleys from the mean line of the surface profile, using conventional equations [76]. AFM data were processed, and statistical analysis of the images was performed using the Gwyddion (2.51) free software.

### 2.8. Cell Cultivation

Adhesive properties of film surfaces and the ability of the films to maintain cell proliferation potential were investigated in experiments with mouse fibroblast NIH 3T3 cells, which were seeded onto films (5 ’ 103 cells/cm^2^) placed in 24-well plates. Fibroblasts were cultured using conventional procedure, in DMEM medium (Gibco, Thermo Fisher Scientific, Inc., Waltham, MA, USA) supplemented with fetal bovine serum, 10%, and a solution of antibiotics (streptomycin 100 µg/mL, penicillin 100 IU/mL) (Gibco, Thermo Fisher Scientific, Inc., Waltham, MA, USA) in a CO_2_ incubator with CO_2_ level maintained at 5%, at a temperature of 37 °C. The medium was replaced every three days.

Viability of cultured fibroblast NIH 3T3 cells was evaluated using 3-(4,5-dimethylthiazol-2-yl)-2,5-diphenyl tetrazolium bromide (MTT) (Sigma-Aldrich, Saint Louis, MO, USA) assay. Viability evaluation was based on the ability of dehydrogenases of living cells to reduce 3-(4,5-dimethylthiazol-2-yl)-2,5-diphenyl tetrazolium bromide to formazan, which characterizes mitochondrial activity, estimates the abundance of living cells, and indirectly indicates the ability of cells to proliferate on the scaffolds. MTT solution (50 µL) and complete nutrient medium (950 µL) were added to each well containing a polymer. After 3.5 h incubation, the medium and MTT were replaced by DMSO (MP Biomedicals, Irvine, CA, USA) to dissolve MTT-formazan crystals. After 30 min, the supernatant was transferred to the 96-well plate, and optical density was measured at wavelength 540 nm, using a Bio-Rad 680 microplate reader (Bio-Rad LABORATORIES Inc., Hercules, CA, USA). The number of cells was determined from the calibration graph.

### 2.9. Statistics

Statistical analysis of the results was performed by conventional methods, using the standard software package of Microsoft Excel. The results were obtained by studying 3–4 specimens, and parameters were measured three times on each specimen. Results of using the high-precision methods X-ray (3%) and AFM (5%) were presented as averages of three measurements of three specimens. Surface porosity (pore sizes and number) measurements were given as arithmetic means and standard deviations. The statistical significance of the number of cells in the MTT assay was determined using a Student’s *t*-test (*p* ≤ 0.05).

## 3. Results

### 3.1. Characterization of Pristine and Laser-Treated Polymer Films

**Figure 1:**
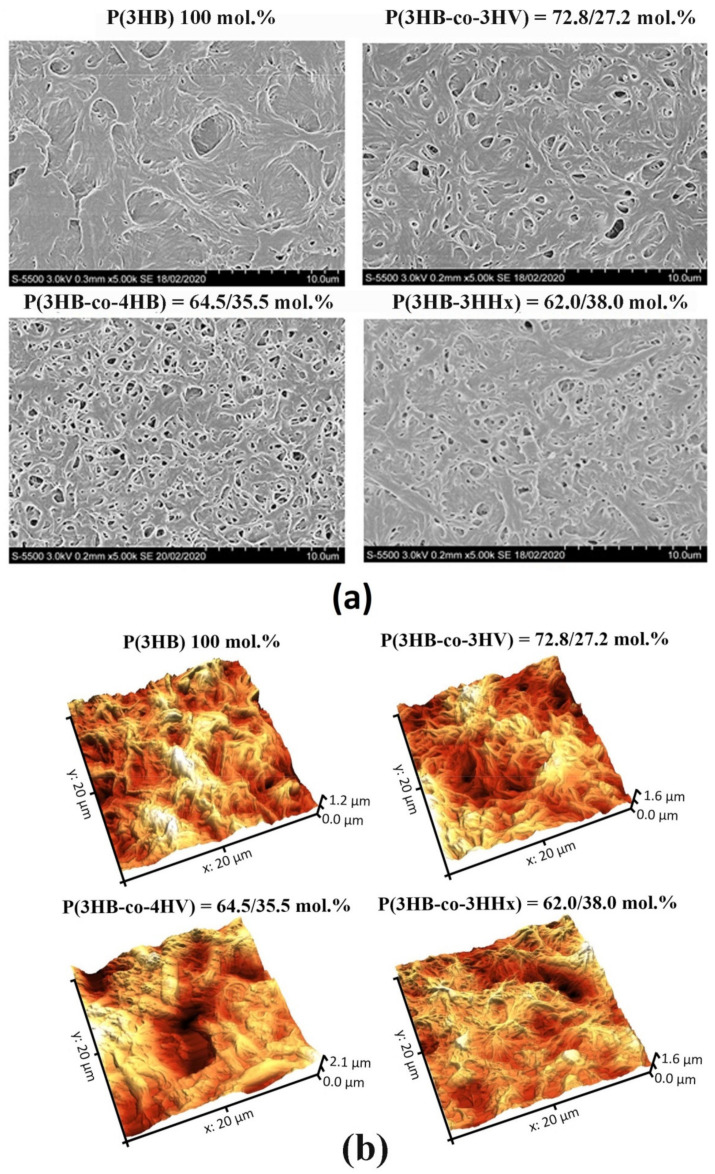
SEM (**a**) and AFM (**b**) images of pristine (non-treated) polymer films prepared from PHAs with different chemical composition.

Films were prepared from four high-purity PHA specimens, which differed in their physicochemical properties (Table 1). Three copolymer specimens, regardless of the second monomer type, had lower weight average molecular weight (Mw) and higher polydispersity (Ð) than the P(3HB) homopolymer: 485–660 kDa and 3.2–3.7, respectively. The corresponding values of the P(3HB) specimen were 920 kDa and 2.5, respectively. The melting point (T_melt_) of P(3HB) was 176 °C and thermal degradation temperature (T_degr_) 280.2 °C. T_melt_ and T_degr_ of the copolymer specimens were lower: 162.5–169.2 °C and 260.1–275.9 °C, respectively. Crystallinity (C_x_) of all copolymers was lower than the C_x_ of P(3HB) (78%) as well. The P(3HB-co-3HV) and P(3HB-co-3HHx) specimens showed similar amorphous to crystalline phase ratios, and their C_x_ values were 54 and 52%, respectively. In the P(3HB-co-4HB) specimens, the amorphous phase was several times greater than the ordered phase, and their C_x_ was 22%. Examination of the pristine (non-treated) films showed that although all four films had the same thickness (298.94 ± 12.79 µm), their morphology and surface properties differed considerably (Figure 1A,B, Table 2 and Table 3).

All copolymer films exhibited greater porosity than the film of P(3HB), on which there were singly positioned pores (38 pores/mm^2^) of average size 0.077 µm. Numerous pores of various sizes, including large, 1.5–3.0-µm pores, were observed on P(3HB-co-4HB) and P(3HB-co-3HV) films. There were numerous but smaller, 0.5–1.0–1.5-µm, pores on P(3HB-co-3HHx) films. The number of pores varied considerably across different types of films: between a few pores per unit area on P(3HB) films and several dozen or more pores on copolymer films.

Pore density on the P(3HB-co-3HV) films was many times higher than on homopolymer films, reaching 529 pores/mm^2^, and their average size was 0.085 µm. The porosity of P(3HB-co-3HHx) films was somewhat lower: the pore density and average size were 279 pores/mm^2^ and 0.045 µm, respectively. P(3HB-co-4HB) films had the highest porosity: 980 pores/mm^2^ of an average size of 0.164 µm. Thus, solvent-cast films of PHAs with different chemical composition exhibited dissimilar pore densities and sizes. The likely reason for this is different crystallization kinetics during solvent evaporation from the polymers differing in Cx values, which may influence attachment and development of eukaryotic cells.

Therefore, a study was performed to determine the differences in the response of films based on four PHA types to two modes of laser treatment, taking into account the differences in the properties of the pristine films.

Hydrophilic/hydrophobic balance of the surface is a parameter that indirectly characterizes hydrophilicity of the film and affects cell adhesion. The hydrophilic/hydrophobic balance is estimated by measuring contact angle for liquids. Water contact angle of the films prepared from the high-crystallinity P(3HB) reached 92.1°, and the calculated values of surface energy and its dispersion and polar components were 30.8, 28.6, and 2.3 mN/m, respectively. Water contact angles of the copolymer films were smaller, varying between 56.3 and 81.7°, with the surface energy rising to between 41.1 and 67.1 mN/m, which suggested that the surfaces of copolymer films were more hydrophilic (Table 2).

An important parameter of polymer products is physicochemical reactivity of the surface. Nanometer roughness determines protein adhesion, cell attachment, growth, and synthesis of specific proteins. The examination of polymer films using atomic force microscopy showed the effect of the chemical composition of PHAs on surface roughness (Table 3).

Pristine P(3HB) films showed the lowest values of arithmetic mean surface roughness (Sa), root mean square roughness (Sq), and peak-to-valley height (Sz): 144.02, 181.583, and 1241.67 nm, respectively. Arithmetic mean surface roughness (Sa) is similar to root mean square roughness (Sq), and the difference is that it is calculated as total difference modules between the data value and the mean rather than squared difference. Peak-to-valley height (Sz) comprises the full range of values; this is total difference between the profile valleys and peaks (between the lowest valley (Sv) and Sp (the highest peak). This parameter was the highest in all pristine copolymer films.

Depending on the mode of treatment, laser ablation (the process of removing material from the surface by irradiating it with a laser beam) results in the formation of spots considerably differing from the untreated regions: both surface topography may change and rough spots and hollows and even perforations may develop on the surface.

Laser treatment of the polymer films was performed by moderate uniform irradiation of the surface, using CO_2_ laser LaserPro Explorer II (Coherent, Santa Clara, CA, USA.) in two essentially different modes of irradiation. The reason for choosing treatment modes was that the major processes in modification of polymer material surface by laser radiation are vaporization and melting. The process responsible for formation of hollows in the shape of grooves and cuts, pits, etc. causes the hollow to become deeper due to vaporization of the material and wider due to melting of the walls and expulsion of the liquid phase by differential vapor pressure. One treatment mode was continuous wave radiation (melting mode) and the other was quasi-pulsed radiation (vaporization mode). Results of laser treatment of polymer films prepared from four PHA types were investigated for the first time. The films were examined using SEM and AFM microscopy, measurements of water contact angles and calculations of surface properties, and testing of biological compatibility of films in eukaryotic cell culture.

### 3.2. Modification of Polymer Film Surfaces in the Continuous Wave Mode

**Figure 2:**
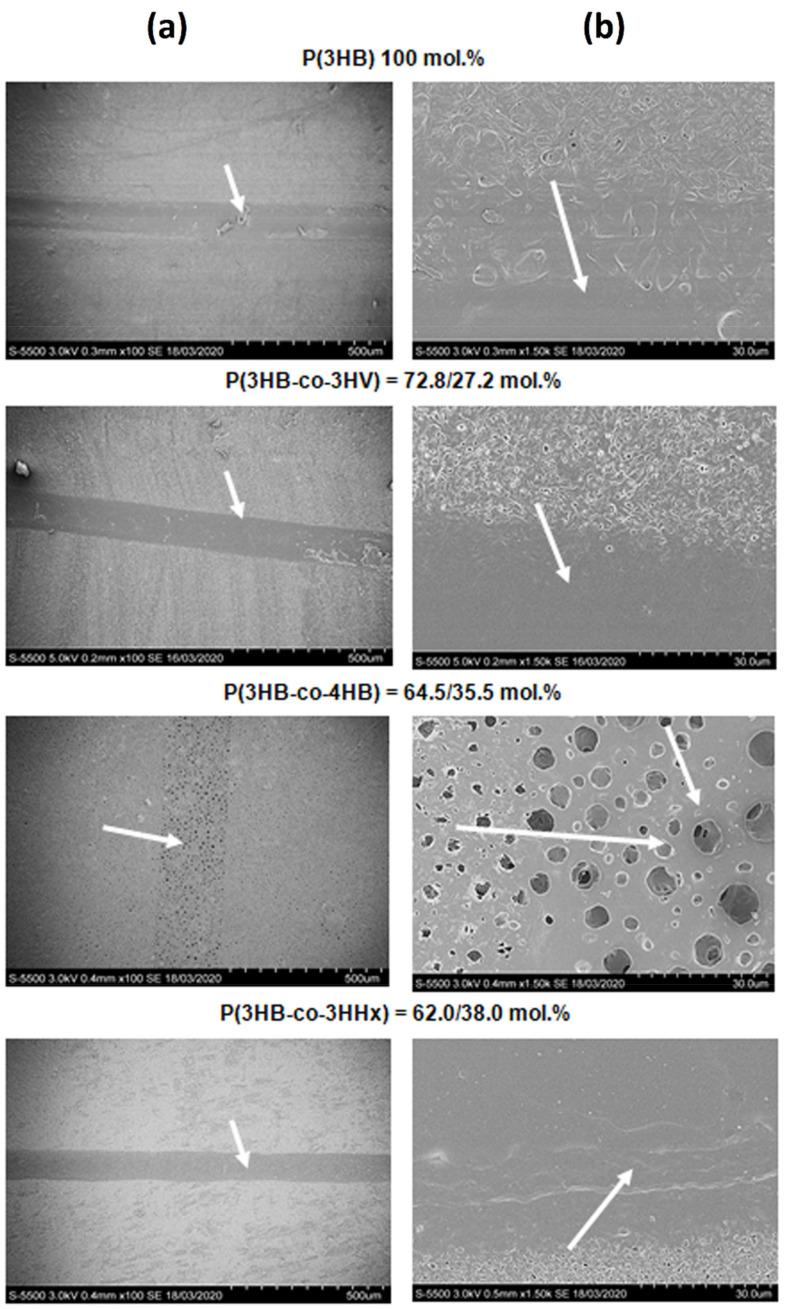
SEM images of laser-treated films prepared from PHAs with different composition. Continuous wave mode. Arrows point at laser-treated and modified regions. Bars: (**a**) = 500 µm, (**b**) = 30 µm.

SEM images of film surfaces in the continuous wave mode are shown in Figure 2. This treatment of the surface of polymer films was performed using CO_2_-laser in the mode of melting along vector lines (with the distance between lines of 1 mm): continuous focused radiation (the beam waist was located on the surface of the treated material); power 10% (3 W), specific power 20,000 (W/m^2^), speed 100% (2 m/s); beam diameter before the focusing lens 2.5 mm and after the focusing lens 0.15 mm. Treatment in this mode causes formation of the melt that cannot be removed by differential vapor pressure, resulting, when the film cools off, in partial sealing of the hollows formed during treatment. Thus, sintered (smoothed) grooves are formed. High-resolution SEM images (bar = 30 µm) show sintered and smoothed regions on the films prepared from PHAs except for the films of P(3HB-co-4HB). On the initially most porous P(3HB-co-4HB) films, numerous pores were formed after laser treatment, and most of them were large (3.0–3.5 µm) (Figure 2).

Significant parameters of PHAs, which determine the conditions of fabricating or processing products from melts, are melting point (T_melt_) and thermal degradation temperature (T_degr_). Therefore, laser treatment procedure should take into account thermal properties of the polymers and differences in these properties between PHAs with dissimilar chemical composition.

**Figure 3:**
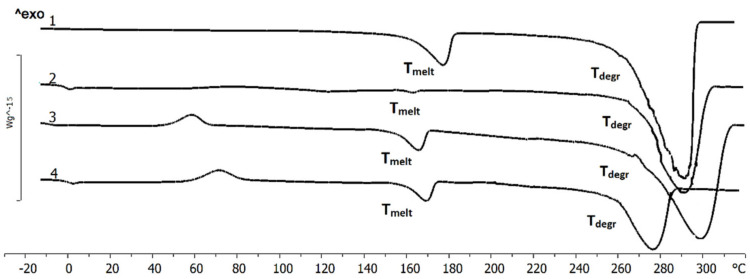
Results of thermal analysis of PHAs with different composition: 1-P(3HB), 2-P(3HB-co-3HV), 3-P(3HB-co-4HB), 4-P(3HB-co-3HHx) (numbering as in Table 1).

The temperatures of the successive phase transitions determined by DSC are provided in Figure 3. The melting range of P(3HB) was 160–185 °C, and the Tmelt was 176 °C. The thermal degradation range of P(3HB) was 275–280 °C. After the specimen was reheated, its melting temperature decreased while its crystallization temperature remained unchanged. When the polymer was heated to its melting point and then maintained at that temperature for 15 min, no change occurred in the position and form of the melting peak. The substantial difference between the temperature of the onset of melting (160 °C) and the temperature of the onset of degradation (260 °C) is fundamentally important for polymer processing, enabling the use of generally accepted methods: solvent casting, extrusion, and injection molding.

Both T_melt_ and T_degr_ of PHA copolymers were somewhat lower. For P(3HB-co-3HHx) and P(3HB-co-4HB), the melting point was lower than the melting peak of the homogenous P(3HB), by 7.1 and 10.5 °C, respectively. The greatest shift of the melting peak relative to P(3HB) was observed for P(3HB-co-3HV): 14 °C. It is important that, although the melting points and the thermal degradation temperatures of PHA copolymers were decreased, the characteristic difference between these parameters remained undiminished, i.e., they retained a valuable property, thermoplasticity.

As PHAs used to prepare films differed in their properties, such as degree of crystallinity, molecular-weight properties, and thermal characteristics (Table 1), the films exhibited different types of melting and different response of the polymer surface to exposure to laser beam. The laser-treated films varied in the width of the grooves or pits formed on the surface of modified regions, distance between them, and total modified area (Table 4).

On the films made from the most thermostable P(3HB), which had the highest Tmelt and Tdegr, the sintered region and the grooves were the narrowest. Thus, the total modified area was the smallest (6.24% of the total film area). All types of copolymer films prepared from PHAs with somewhat lower thermal parameters had wider grooves and larger modified area, which reached 10.7% on the films of P(3HB-co-3HV), 9.07% on the P(3HB-co-4HB) films, and 7.64% on the film of P(3HB-co-3HHx).

Only on the P(3HB) film, measurements of water contact angle in the laser-modified regions showed a 12° decrease in this parameter with a similar increase in surface energy and its dispersion and polar components (Table 2). On the P(3HB-co-4HB) film, these parameters remained almost unchanged, and on the P(3HB-co-3HV) and P(3HB-co-3HHx) films, they increased, indirectly indicating higher hydrophobicity of the modified regions.

**Figure 4:**
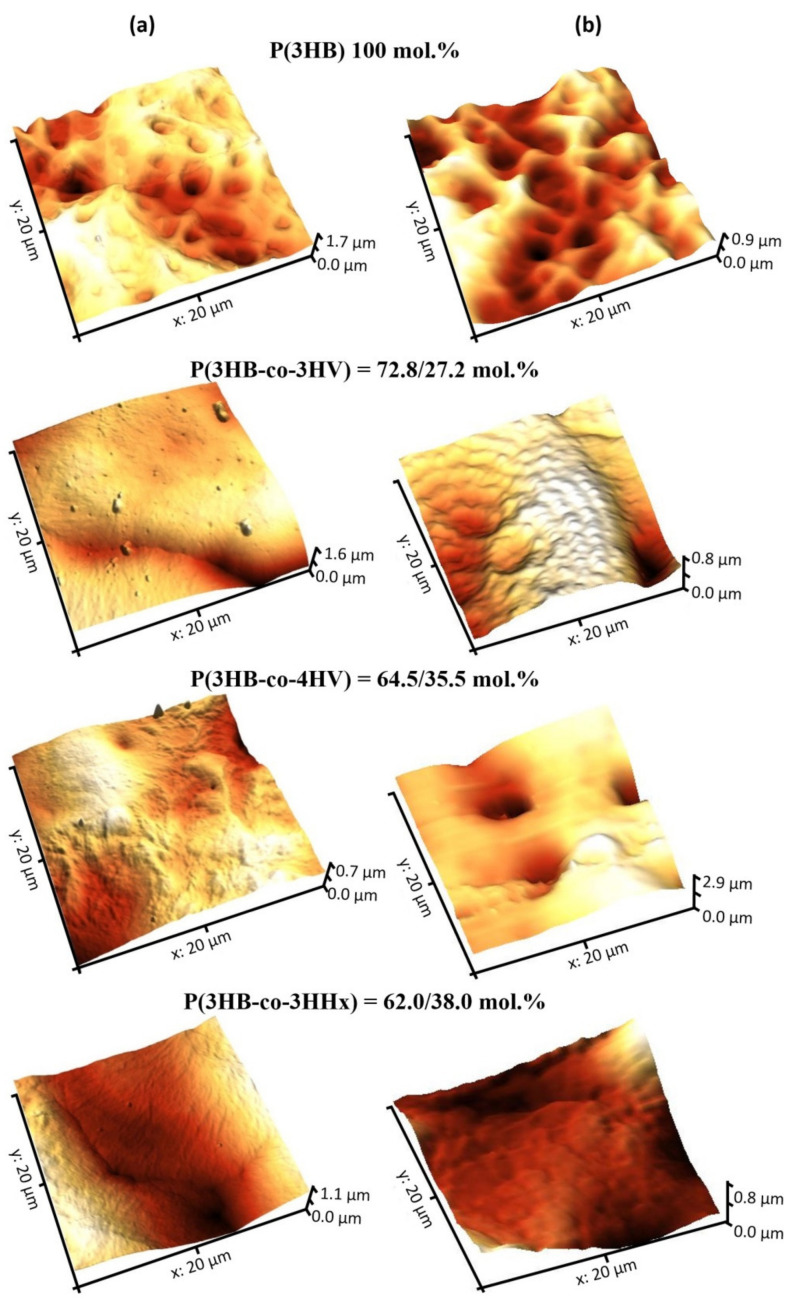
AFM images of surfaces of the laser-treated films prepared from PHAs with different composition: (**a**)—continuous wave mode; (**b**)—quasi-pulsed mode.

Laser treatment in the continuous wave mode produced a pronounced effect on the film surface roughness (Figure 4, Table 3). P(3HB) and P(3HB-co-3HHx) films showed increased arithmetic mean surface roughness (S_a_) and root mean square roughness (S_q_); the other two specimens, with the sintered smoothed regions formed after laser treatment, had lower S_a_, S_q_, and peak-to-valley height (S_z_). The greatest, almost threefold, decrease in these parameters (S_a_; S_q_; S_z_) was observed for the P(3HB-co-4HB) films. Thus, laser treatment in the continuous wave focused mode caused partial sintering of some of the copolymer films, which resulted in smoother surfaces with lower roughness.

### 3.3. Modification of Polymer Film Surfaces in the Quasi-Pulsed Mode

This type of laser treatment was performed in the raster mode (point by point, with the distance between points of 1 mm): quasi-pulsed focused radiation, beam diameter before the focusing lens 2.5 mm and after the focusing lens—0.15 mm; power 45% (13.5 W), specific power 90,000 W/m^2^, speed 50% (1 m/s). Polymer films were treated in the vaporization mode. In this mode, the temperature of the material in the exposed region is higher than its melting point, and material is removed in the form of mist. This mode requires the highest specific power consumption, and it is usually performed in the pulsed or quasi-pulsed laser mode.

**Figure 5:**
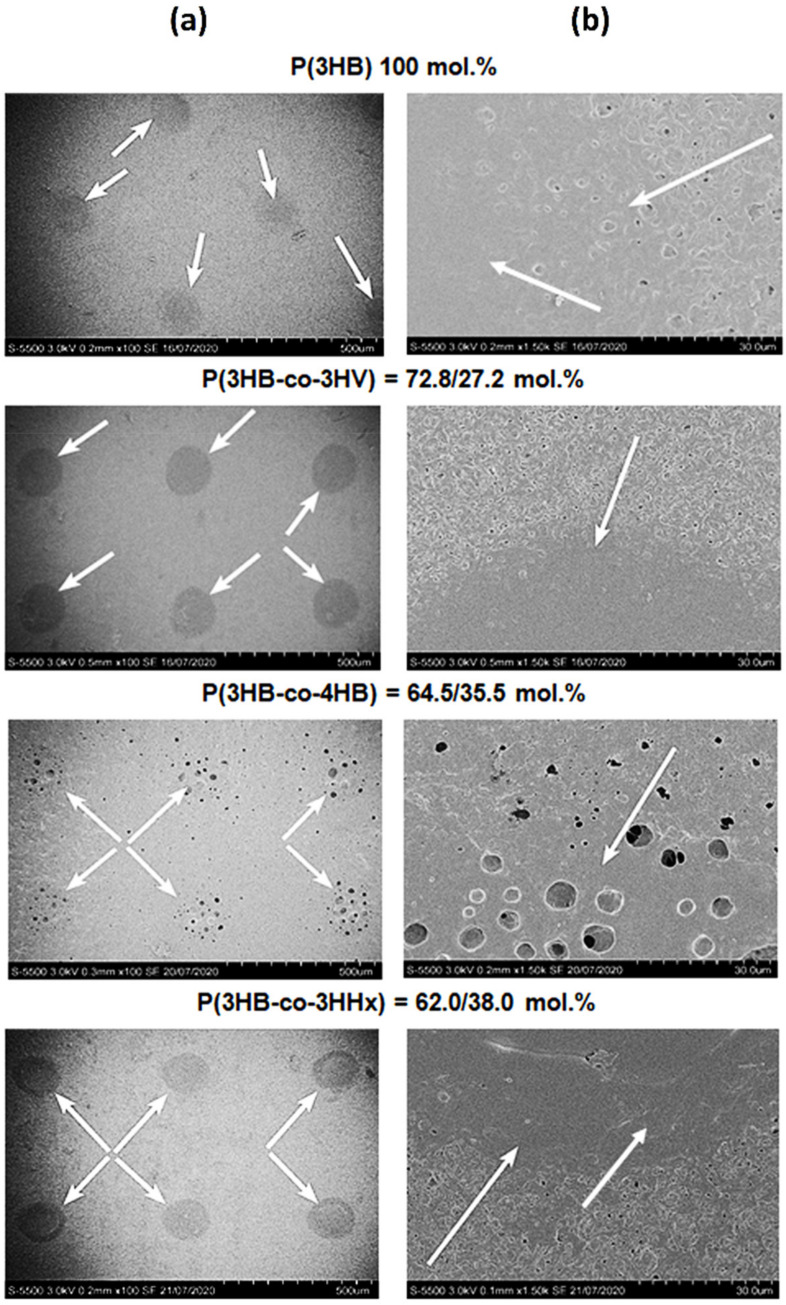
SEM images of laser-treated films prepared from PHAs with different composition. Quasi-pulsed mode. Arrows point at laser-treated and modified regions. Bars: (**a**) = 500 µm, (**b**) = 30 µm.

The amount of power was chosen based on the preliminary study, which showed that at a power below 13 W, no ablation of material occurred, and at a power above 15 W, the material was perforated, and that was undesirable. SEM images show (Figure 5) that pits were formed on the surfaces of all films. The treatment in the quasi-pulsed mode, like the treatment in the continuous wave mode, caused more significant sintering of the copolymer films, on which larger-diameter pits were formed, and their area and the total modified area were greater and inter-pit spacing was smaller than on the more thermostable P(3HB) films (Table 4). Like in the experiment with continuous wave treatment, on the initially most porous films of P(3HB-co-4HB), numerous large (from 2 to 3.0–3.5 µm) pores were formed.

Changes caused by quasi-pulsed laser radiation in the film surface properties are shown in Table 2 and Table 3. The values of water contact angles are the averaged data obtained by measuring not only modified regions (pits) but also non-treated inter-pit areas. The reason for this is that water drops were considerably larger than the pits, and one drop usually covered 4–5 pits and the surface between them.

The effect of quasi-pulsed radiation differed from the effect of continuous wave radiation. A more considerable decrease in water contact angle, to 67.7°, was observed on P(3HB) films, and the increase in the surface energy and polar component on those films was more pronounced as well. Moreover, the surface of P(3HB-co-4HB) films became more hydrophilic as the water contact angle decreased and the surface energy and polar component increased, in contrast to the changes observed after continuous wave treatment. The values of water contact angle of P(3HB-co-3HV) and P(3HB-co-3HHx) films increased after both continuous wave and quasi-pulsed treatments.

Whereas continuous wave radiation smoothed the modified surface and decreased roughness parameters of half of the films, quasi-pulsed treatment reduced arithmetic mean surface roughness and root mean square roughness of P(3HB-co-3HV), P(3HB-co-4HB), and P(3HB-co-3HHx) films but slightly increased roughness parameters of the P(3HB) films (Table 3).

### 3.4. Biological Properties of the Pristine and Laser-Treated Films Prepared from PHAs with Different Composition

Biocompatibility of cell scaffolds is to a great extent determined by physicochemical reactivity of their surface. The main factors that regulate cell growth and function are scaffold surface topography, roughness, structure, and chemical and phase compositions. The initial behavior of the cells on the surface largely determines subsequent processes of cell differentiation and proliferation.

**Figure 6:**
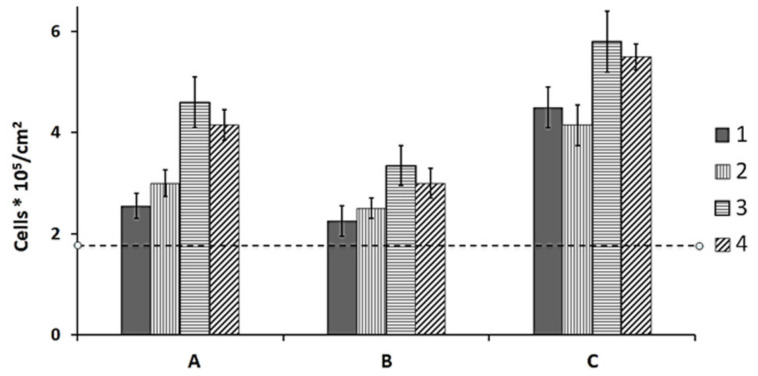
Numbers of viable cells in MTT assay in the 6-day-old culture of NIH 3T3 fibroblasts on pristine and laser-treated films of PHAs with different composition: 1-P(3HB); 2-P(3HB-co-3HV); P(3HB-co-4HB); P(3HB-co-3HHx); A—pristine (non-treated) films; B—continuous wave mode, C—quasi-pulsed mode.

None of the films, regardless of the PHA composition and treatment mode, had any negative effect on the functional properties of cells relative to the control (polystyrene). None of the films, including laser-treated ones, exhibited cytotoxicity to the NIH 3T3 mouse fibroblast cells. However, the number of viable cells determined in the MTT assay differed depending on both PHA composition and laser treatment mode (Figure 6).

In the 6-day cell culture on the pristine P(3HB) and P(3HB-co-3HV) films, there were 2.55–3.00*105 fibroblasts/cm^2^, and this number was statistically significantly higher than the number of cells on polystyrene (control) (Figure 6A). Larger cell counts (4.25 and 4.6*10^5^/cm^2^), the difference between which was not statistically significant, were observed on the films of copolymers that contained 3HHx and 4HB as second monomers and had the lowest degrees of crystallinity: 52% and 22%, respectively.

Continuous wave radiation, which caused the formation of sintered spots on the film surface, and thus increased surface hydrophobicity, adversely affected cell attachment. Therefore, the number of viable fibroblasts was significantly lower on the films treated in that mode compared to non-treated films (Figure 6B). The number of cells on the P(3HB) and P(3HB-co-3HV) films was comparable to the cell number on polystyrene (control), and the number of cells on the P(3HB-co-4HB) and P(3HB-co-3HHx) films was somewhat higher. Hence, although continuous wave radiation caused polymer sintering, the biological compatibility of the modified films and NIH 3T3 mouse fibroblast cells was not dramatically impaired.

Treatment in quasi-pulsed mode, which formed pits on the film surface, improved the adhesive properties of the films, including water contact angle, surface energy, and roughness parameters. Films treated in the quasi-pulsed mode, which caused vaporization of the melt polymer from the surface and formation of pits, were the most favorable for proliferation of NIH 3T3 fibroblasts. Their number was greater than on pristine films, reaching 4.0–4.5*10^5^/cm^2^ on P(3HB) and P(3HB-co-3HV) films and 5.5–5.8*10^5^/cm^2^ on P(3HB-co-3HHx) and P(3HB-co-4HB) films (Figure 6C).

## 4. Discussion

The development of science and technology widens the applications of synthetic and natural high molecular-weight compounds. The structure and mechanical/physical properties of the polymers and polymer-based products are determined by their intended applications (construction, agriculture, municipal engineering medicine, etc.).

A promising approach to modification of polymers and polymer-based products is laser treatment using lasers generating different radiation types. The most powerful and well developed for practical applications are CO_2_ gas discharge lasers [65,67,71] while lasers generating ultrashort pulses modify surfaces with high precision and produce minimal thermal and chemical effects on polymers [55,77]. Laser radiation is supposed to create spots that structurally differ from the pristine material and may have enhanced adhesive properties [63]. This effect was observed in experiments with laser-treated synthetic and natural polymers, including degradable polyhydroxyalkanoates (PHAs), tested in the present study.

In the present study, for the first time, four PHA types with different chemical composition were treated using CO_2_ laser in the continuous wave and quasi-pulsed modes, with varied treatment power and speed. Examination of the laser-treated films showed that changes in their surface properties were not the same and were determined by both the mode of laser treatment and the composition and properties of the films.

Films prepared from four PHA types had considerably different surface roughness parameters and hydrophobic/hydrophilic balance (water contact angle) even before laser treatment. Water contact angle and surface energy of P(3HB) films were 92° and 30 mN/m, respectively, and roughness was 154 nm. The corresponding parameters of all copolymer films differed from those of P(3HB): water contact angle decreased to 80–56°, surface energy increased to 41–57 mN/m, and roughness increased to 172–290 nm. A number of studies demonstrated a similar increase in PHA hydrophilicity in polymers with longer carbon chain (the so-called medium-chain-length polymers such as copolymers containing 3HHx) [78,79]. Surguchenko et al. [80] reported the data consistent with our results: film surface roughness increased because of incorporation of 3HV monomers into the 3HB chain. There are, however, other data showing that films produced from P(3HB-co-4HB) with a higher percentage of 4HB had a smoother surface, with lower roughness parameters [1,81]. Even slight alterations of surface profile may cause very diverse changes in response of the cells, from an insignificant increase in the activity of cells to considerable inhibition thereof. Cells, however, differ in their sensitivity to variations in surface roughness and topography.

Thus, films of PHAs with different composition had dissimilar properties even before laser treatment. Laser treatment of the films was performed by moderate uniform irradiation of the surface, using CO_2_ laser LaserPro Explorer II in two essentially different modes of irradiation. Continuous wave irradiation resulted in formation of sintered grooves on the film surface. Treatment by quasi-pulsed radiation was performed in the raster mode (point by point) and resulted in the formation of pits without pronounced sintered regions on the surface of all film types. Films treated in either mode showed differences in surface modification, which were associated with their chemical composition.

Laser-treated P(3HB) films exhibited a decrease in water contact angle, which was more significant after the treatment in quasi-pulsed mode; roughness parameters were changed by the treatment in both modes. The effect of laser treatment on surface morphology and roughness was reported for a variety of polymers. For example, laser-treated carbon-coated polyethylene films exhibited increased wettability and roughness [60]; CO_2_-laser texturing of poly(L-lactide) films influenced surface microhardness, roughness, and wettability [61]; femtosecond laser modification of thin chitosan films increased surface roughness from 0.5 to almost 3.0 µm.

The search of literature showed that in most studies devoted to laser treatment of PHAs, the authors used homopolymer P(3HB). One of the first studies [64] demonstrated that treatment of P(3HB) products using a nitrogen-blanketed CO_2_-laser resulted in the formation of 60 to 100 µm deep regions with the structure different from the non-treated surfaces. Authors of another study [65] developed a method of interaction between laser radiation and P(3HB) and, using a CO_2_-laser, produced films with surfaces modified to different degrees (from changes in roughness to formation of holes), which had enhanced hydrophilicity, determined by decreased water contact angles. Flexible P(3HB) films were treated using LaserPro Explorer and LaserPro Spirit CO_2_-lasers [66] at varied power and speed in the focused and defocused modes. Water contact angle of the films was decreased in the focused and defocused modes at the processing speed of 0.8 m/s and 1.8 m/s and power 9 W and 12 W, respectively. In most treatments, surface free energy rose insignificantly (10–16%) but its polar component increased considerably, by a factor of 3–5, especially when radiation power was increased.

Michaljanicova et al. [67] reported a study in which P(3HB and polylactide were treated using KrF and ArF excimer lasers. Under the same treatment conditions, the ArF laser caused more considerable changes in the surface chemical composition and morphology. The treatment of P(3HB) with KrF laser caused the opposite effect on surface morphology compared to PLA. Low laser energy applied (up to 15 mJ cm^−2^) did not affect the material surface significantly, but with higher laser fluence, the roughness increased rapidly. An increase in the number of pulses during the modification caused a significant increase in P(3HB) roughness. In another study [68], the surfaces of P(3HB) and other polymer (PLA, poly(methyl methacrylate, and polyurethane) films were modified using a krypton fluoride excimer laser, which was employed to produce cavities and orderly perforated holes on the films. The modified films were considered as suitable for tissue engineering purposes.

Treatment of P(3HB-co-3HV) films in continuous wave and quasi-pulsed modes resulted in a decrease in surface roughness, an increase in water contact angle, and a decrease in surface energy, which indirectly indicated an increase in hydrophobicity of the modified regions. In a number of studies, films of this copolymer were treated using Nd:YAG and KrF excimer lasers. However, 3HV content of P(3HB-co-3HV) was much lower compared to the present study (35.5 mol.%). Treatment of the films of P(3HB-co-3HV) containing 3HV 11 mol.% with the Nd:YAG laser resulted in formation of micropores shaped as distinct oval cavities of size 150×100 µm, area 11.7 × 10^−3^ mm^2^, and distance between them up to 200 µm [70]. Similar films of the copolymer containing 3HV 11 mol.% were perforated by ultraviolet laser ablation using the fourth harmonic of a Nd:YAG laser [71]. In the laser-treated films, which were 30 µm thick, 100-µm-diameter pores were formed at equal distances of 200 µm. The treatment neither reduced flexibility of the films nor decreased their mechanical strength. IR spectra did not reveal any significant amounts of copolymer degradation products at the micropore edges, but these regions were less crystalline. The authors believe that amorphization of the copolymer material caused by laser treatment will facilitate cell attachment. In another study [72], films of P(3HB-co-3HV) with 3HV 8 mol.% were modified using KrF laser and method was also supplemented by treatment with Ar+ plasma. Samples were exposed to the laser treatment with different numbers of laser pulses (from 1000 to 6000), which resulted in a decrease in water contact angles from 60–68° to 40–54°. Treatment of the films by both laser and plasma cause considerable changes of the surface. By increasing the density of laser fluence without changing the number of laser pulses, the authors managed to increase P(3HB-co-3HV) surface roughness considerably (31.9 → 47.1 → 270.0 nm). Modification of surface by laser with a high number of pulses and fluence led to creation of surface layers with huge valleys and very high roughness. These structures were caused by extreme effect of ablation in combination with mass transfer.

Quasi-pulsed irradiation of another type of the films, prepared from the copolymer with the lowest crystallinity, P(3HB-co-4HB) (C_x_ 22%), and, thus, initially most porous ones, resulted in hydrophilization of the film surface, i.e., a decrease in water contact angle and an increase in surface energy and its polar component, contrary to the results of continuous wave irradiation. Laser treatment of specimens made from P(3HB-co-4HB), regardless of the mode employed, induced formation of new numerous large pores (of diameter up to 3.0–3.5 µm) in the modified regions (grooves and pits), in contrast to all other films. The two treatment modes produced different effects on roughness parameters. Under continuous wave radiation, surface roughness parameters were considerably reduced, but under quasi-pulsed radiation, they were similar to those of the pristine films. No data on laser treatment of P(3HB-co-4HB) films could be found in the available literature.

Continuous wave irradiation of another type of the films, prepared from medium-chain-length PHA, which contained 3HHx monomers, caused some increase in the arithmetic mean surface roughness and root mean square roughness while quasi-pulsed irradiation of the films of this composition resulted in an insignificant decrease in surface roughness. Both treatment modes induced an increase in water contact angle and a decrease in surface energy and its dispersion and polar components. Ortiz et al. [28] described picosecond laser ablation of medium-chain-length polymer, but that was poly(3-hydroxy octanoate-co-3-hydroxy decanoate)—neat and blended with P(3HB). In that study, the authors for the first time used picosecond pulsed laser ablation to microstructure the surface of P(3HO-3HD) films and compared them with P(3HB) and P(3HB)/P(3HO-3HD) blend. Picosecond pulses changed topography of the three specimens, which initially differed in their thermal and mechanical properties, but produced an insignificant effect on the chemical and microstructural properties of the polymer. These results suggested photochemical ablation as the dominant mechanism during picosecond laser treatment of the PHA films used in that study, especially at a wavelength of 355 nm. Laser treatment resulted in formation of grooves and caverns on the film surface, and their depth and size differed depending on the material type and treatment mode. It is important that surface modification was performed in one-step process with minimal thermal impact on the surface of the polymer. Similar modification of the surface of poly(L-lactide) thin films exposed to CO_2_-laser radiation was described in a study by Kobielarz et al. [82]: the authors observed formation of a characteristic distinct pattern of parallel and equidistant grooves and ridges that repeated over a distance of 40–60 µm. Another study [83] demonstrated surface modification of poly(L-lactide)/hydroxyapatite films using femtosecond laser and formation of 10–50-µm grooves, which enlarged surface area. Formation of a wrinkled structure and changes in the surface roughness of the polystyrene films doped with acetylsalicylic acid and irradiated using excimer laser (krypton fluoride, a wavelength of 248 nm) were reported in [84].

The advantages of laser treatment as one of the various methods of shape forming and surface modification of biodegradable polymers for biomedical applications include its ability to process complex-shaped surfaces without using toxic chemical components. Lasers afford the possibility of achieving high spatial controllability and high-precision structuring, which is expected to help construct complex biomedical devices with good biocompatibility [85]. Laser interaction, which results in surface modification, is unique, causing changes in polymer properties such as resistance to wear and surface modification [86]. Therefore, alteration of chemistry and morphology of polymer surface by laser treatment has been increasingly used to develop biocompatible materials and biomedical implants for enhancing protein and cell attachment.

The study of biological properties of the films of four PHA types treated in the modes of continuous wave and quasi-pulsed laser radiation revealed opposite effects of the two treatment modes on the development of NIH 3T3 mouse fibroblasts. The number of cells on the films treated using quasi-pulsed radiation increased considerably compared to the pristine films and films treated in the continuous wave mode: by a factor of 1.26–1.76 and 1.66–1.83, respectively. There are similar literature data suggesting the favorable effect produced by laser modification of the surfaces of the films fabricated from some PHAs on attachment and proliferation of various eukaryotic cells. Laser modification of the films of P(3HB) and PLA, PMMA, and PU/PDMS using a krypton fluoride excimer laser enhanced adhesion and proliferation of human fibroblasts [61]. Films of poly(3-hydroxybutyrate-co-3-hydroxyvalerate) processed to insert high-density micropores through a Nd:YAG laser ablation process provided firmer attachment of keratinocytes and enhanced migration of the cells through micropores compared to pristine films [70]. Flexible and biodegradable microperforated P(3HB-co-3HV) films prepared by ultraviolet laser ablation enabled proliferation of immortal human keratinocytes [71]. A study by Slepička et al. [72] described the successful use of krypton fluoride (KrF) excimer laser to treat films of P(3HB-co-3HV) with a minor fraction of 3HV (8 mol.%). Surface modification resulted in formation of large hollows and, thus, a dramatic increase in roughness parameters. Good results were obtained in the tests of the response of mouse embryonic fibroblasts (NIH 3T3) and human bone osteosarcoma (U-2 OS) cells. Similar results were described for other polymer types. Femtosecond laser treatment favorably influenced attachment and orientation of mouse calvaria osteoblasts and human adipose derived mesenchymal stem cells on thin films of pure chitosan and composite blends of chitosan (Ch)/HAp/ZrO_2_ [62]. The authors of another work [83] studied human osteoblasts ATCC cultured on the surface of poly(L-lactide)/hydroxyapatite films modified using femtosecond laser and did not detect any cytotoxicity. Therefore, they concluded that laser treatment could be regarded as a promising method to modify scaffolds for tissue engineering and facilitate integration of a bioresorbable implant and bone. A study by Takayama et al. [87] showed that femtosecond laser created micro through-holes in biodegradable PLLA films, enhanced adhesion of myoblasts, and facilitated their proliferation and differentiation. ChR2-C2C12 and UT-C2C12 cells were seeded onto films with micro through-holes, each of which was created by a single femtosecond laser pulse. Cell adhesion was enhanced on films with the holes produced by laser irradiation. Furthermore, cell proliferation occurred at a higher rate on films with micro through-holes, which penetrated the film, compared with the films with micro crates, which did not penetrate the film. Cell differentiation was accelerated, and cell alignment was high on structures with the width of 20–30 µm and on films with 100 µm between single arrays.

In contrast to quasi-pulsed irradiation, on the films of all PHA types treated using continuous wave mode, which caused the formation of sintered regions on film surface, the number of viable fibroblasts was 13.0–27.2% lower (depending on the film type) than on pristine films. This is an important result, offering an opportunity for the targeted surface modification of polymer products aimed at preventing or facilitating cell attachment: e.g., to reduce formation of biofilm on plastic food packaging and vice versa, to stimulate development of cell cultures on films used as cell scaffolds in cellular engineering technologies.

## 5. Conclusions

The present study was the first to investigate the properties of the solvent cast films of PHAs with different composition, which were modified using laser irradiation in different modes. CO_2_-laser was used in continuous wave (3 W; 2 m/s) and quasi-pulsed (13.5 W; 1 m/s) modes to treat films prepared by solvent casting technique from four PHAs types, i.e., poly-3-hydroxybutyrate and three copolymers of 3-hydroxybutyrate: with 4-hydroxybutyrate, 3-hydroxyvalerate, and 3-hydroxyhexanoate (each second monomer constituting about 30 mol.%). The PHAs differed in their thermal and molecular weight properties and degree of crystallinity. Pristine films differed in porosity, hydrophilicity, and roughness parameters. The two modes of laser treatment altered these parameters and biocompatibility in diverse ways. Films of P(3HB) had water contact angle and surface energy of 92° and 30.8 mN/m, respectively, and average roughness of 144 nm. The water contact angle of copolymer films decreased to 80–56°, and surface energy and roughness increased to 41–57 mN/m and 172–290 nm, respectively.

Treatment in either mode resulted in different modifications of the films, depending on their composition and irradiation mode. Laser-treated P(3HB) films exhibited a decrease in water contact angle, which was more considerable after the treatment in quasi-pulsed mode. Roughness parameters were changed by the treatment in both modes. Continuous wave line-by-line irradiation caused formation of sintered grooves on the film surface, which exhibited some change in water contact angle (76–80°) and reduced roughness parameters (to 40–45 mN/m) for most films. Treatment in the quasi-pulsed raster mode resulted in the formation of the pits with no pronounced sintered regions on the film surface, a more considerable decrease in water contact angle (to 67–76°), and increased roughness of most specimens.

A colorimetric assay for assessing cell metabolic activity (MTT) in NIH 3T3 mouse fibroblast culture showed that the number of fibroblasts on the films treated in the continuous wave mode was somewhat lower. The treatment in quasi-pulsed radiation mode caused an increase in the number of viable cells by a factor of 1.26–1.76, depending on PHA composition. This is an important result, offering an opportunity of targeted surface modification of PHA products aimed at preventing or facilitating cell attachment.

New data were obtained on the effects of laser treatment of the promising group of biodegradable polymers.

## Figures and Tables

**Table 1:** Composition and physicochemical properties of different PHAs.

Specimen No.	PHA Composition	Monomer Ratios, mol.%	M_w_, kDa	Ð	C_x_, %	T_melt_, °C	T_degr_, °C
1	P(3HB)	100.0	920	2.5	78	176.3	280.2
2	P(3HB-co-3HV)	72.8/27.2	576	3.2	54	162.5	275.9
3	P(3HB-co-4HB)	64.5/35.5	660	3.6	22	165.5	278.4
4	P(3HB-co-3HHx)	62.0/38.0	486	3.7	52	169.2	260.1

**Table 2:** Surface properties of the pristine and laser-treated films prepared from PHAs with different composition.

PHA Composition, mol.%	Water Contact Angle,°	Surface Energy, mN/m	Dispersion Component, mN/m	Polar Component, mN/m
**Pristine films**				
P(3HB) = 100.0	92.1 ± 6.33	30.8 ± 0.53	28.6 ± 0.31	2.3 ± 0.21
P(3HB-co-3HV) = 72.8/27.2	69.4 ± 9.4	50.8 ± 2.64	43.7 ± 1.46	7.1 ± 1.18
P(3HB-co-4HB) = 64.5/35.5	81.7 ± 3.24	41.4 ± 0.89	37.8 ± 0.7	3.7 ± 0.19
P(3HB-co-3HHx) = 620/38.0	56.3 ± 6.16	57.1 ± 2.89	43.5 ± 2.03	13.6 ± 0.85
**Continuous wave mode**				
P(3HB) = 100.0	80.5 ± 2.5	40.8 ± 0.52	39.9 ± 0.31	4.9 ± 0.20
P(3HB-co-3HV) = 72.8/27.2	76.2 ± 2.6	46.0 ± 3.22	41.1 ± 2.64	4.9 ± 0.58
P(3HB-co-4HB) = 64.5/35.5	79.9 ± 4.24	42.0 ± 1.15	37.7 ± 0.89	4.3 ± 0.26
P(3HB-co-3HHx) = 620/38.0	70.0 ± 5.25	45.5 ± 1.29	36.8 ± 0.86	8.7 ± 0.42
**Quasi-pulsed mode**				
P(3HB) = 100.0	67.7 ± 3.30	48.6 ± 1.14	39.7 ± 0.58	9.0 ± 0.56
P(3HB-co-3HV) = 72.8/27.2	84.6 ± 3.75	41.7 ± 1.49	39.2 ± 1.12	2.5 ± 0.37
P(3HB-co-4HB) = 64.5/35.5	68.2 ± 2.36	47.5 ± 1.13	38.3 ± 0.69	9.1 ± 0.44
P(3HB-co-3HHx) = 620/38.0	76.4 ± 3.29	44.3 ± 2.28	39.0 ± 1.72	5.3 ± 0.57

**Table 3:** Surface roughness parameters of the pristine and laser-treated films prepared from PHAs with different composition, based on results of atomic force microscopy (AFM).

PHA Composition, mol.%	Arithmetic Mean Surface Roughness (Sa) nm	Root Mean Square Roughness (Sq) nm	Peak-to-Valley Height (Sz) nm
**Pristine films**			
P(3HB) = 100.0	144.02	181.583	1241.67
P(3HB-co-3HV) = 72.8/27.2	209.136	255.722	1577.85
P(3HB-co-4HB) = 64.5/35.5	281.721	355.325	2135.25
P(3HB-co-3HHx) = 620/38.0	175.743	224.334	1648.50
**Continuous wave mode**			
P(3HB) = 100.0	232.454	282.227	1682.01
P(3HB-co-3HV) = 72.8/27.2	152.053	202.653	1437.41
P(3HB-co-4HB) = 64.5/35.5	83.408	106.279	671.592
P(3HB-co-3HHx) = 620/38.0	216.518	251.907	1079.69
**Quasi-pulsed mode**			
P(3HB) = 100.0	162.586	192.941	973.004
P(3HB-co-3HV) = 72.8/27.2	91.728	123.714	814.021
P(3HB-co-4HB) = 64.5/35.5	275.825	442.297	5135.20
P(3HB-co-3HHx) = 62.0/38.0	120.839	159.291	1079.35

**Table 4:** Characterization of the surface structural elements of laser-treated films prepared from PHAs with different composition.

**Continuous Wave Mode**					
**PHA Composition, mol.%**	**Width of Grooves, µm**		**Distance between Grooves, µm**		**Modified Area, %**
P(3HB) = 100.0	115.75 ± 5.61		890.18 ± 5.30		11.56 ± 1.03
P(3HB-co-3HV) = 72.8/27.2	140.19 ± 2.94		891.34 ± 5.85		12.15 ± 1.14
P(3HB-co-4HB) = 64.5/35.5	163.27 ± 6.52		864.17 ± 7.82		15.68 ± 0.67
P(3HB-co-3HHx) = 620/38.0	125.10 ± 3.80		889.56 ± 1.87		12.99 ± 0.95
**Quasi-Pulsed Mode**					
**PHA Composition, mol.%**	Pit Diameter Calculated from the Formula d = 2*√S/π, µm	**Pit Area, µm^2^**		**Distance between Pits, µm**	**Modified Area, %**
P(3HB) = 100.0	160.12	20,126.58 ± 1327.99		342.42 ± 18.56	6.24 ± 0.49
P(3HB-co-3HV) = 72.8/27.2	187.57	27,618.06 ± 2679.87		322.17 ± 21.32	10.07 ± 1.06
P(3HB-co-4HB) = 64.5/35.5	172.00	23,224.50 ± 2457.80		335.87 ± 17.72	9.07 ± 0.61
P(3HB-co-3HHx) = 62.0/38.0	198.93	31,063.36 ± 4611.14		338.89 ± 24.05	7.64 ± 1.01

## Data Availability

Not applicable.
