# Correction: Volova et al. Laser Processing of Polymer Films Fabricated from PHAs Differing in Their Monomer Composition. *Polymers* 2021, *13*, 1553

**DOI:** 10.3390/polym16081075

**Published:** 2024-04-12

**Authors:** Tatiana G. Volova, Alexey I. Golubev, Ivan V. Nemtsev, Anna V. Lukyanenko, Alexey E. Dudaev, Ekaterina I. Shishatskaya

**Affiliations:** 1 Basic Department of Biotechnology, School of Fundamental Biology and Biotechnology, Siberian Federal University, 79 Svobodnyi Av., 660041 Krasnoyarsk, Russia; 2 Institute of Biophysics SB RAS, Federal Research Center “Krasnoyarsk Science Center SB RAS”, 50/50 Akademgorodok, 660036 Krasnoyarsk, Russia; 3 L.V. Kirensky Institute of Physics SB RAS, Federal Research Center “Krasnoyarsk Science Center SB RAS”, 50/38 Akademgorodok, 660036 Krasnoyarsk, Russia; 4 Special Design and Technological Bureau ‘Nauka’ Federal Research Center “Krasnoyarsk Science Center SB RAS”, 50/45 Akademgorodok, 660036 Krasnoyarsk, Russia; 5 Federal Research Center “Krasnoyarsk Science Center of the Siberian Branch of the Russian Academy of Sciences” 50 Akademgorodok, 660036 Krasnoyarsk, Russia

In the original publication [1], there was a mistake in Figure 1 as published. **The image of the sample P(3HB-co-3HV) = 72.8/27.2 mol.% is mixed up.** The corrected Figure 1 appears below. The authors apologize for any inconvenience caused and state that the scientific conclusions are unaffected. This correction was approved by the Academic Editor. The original publication has also been updated.

## Figures and Tables

**Figure 1:**
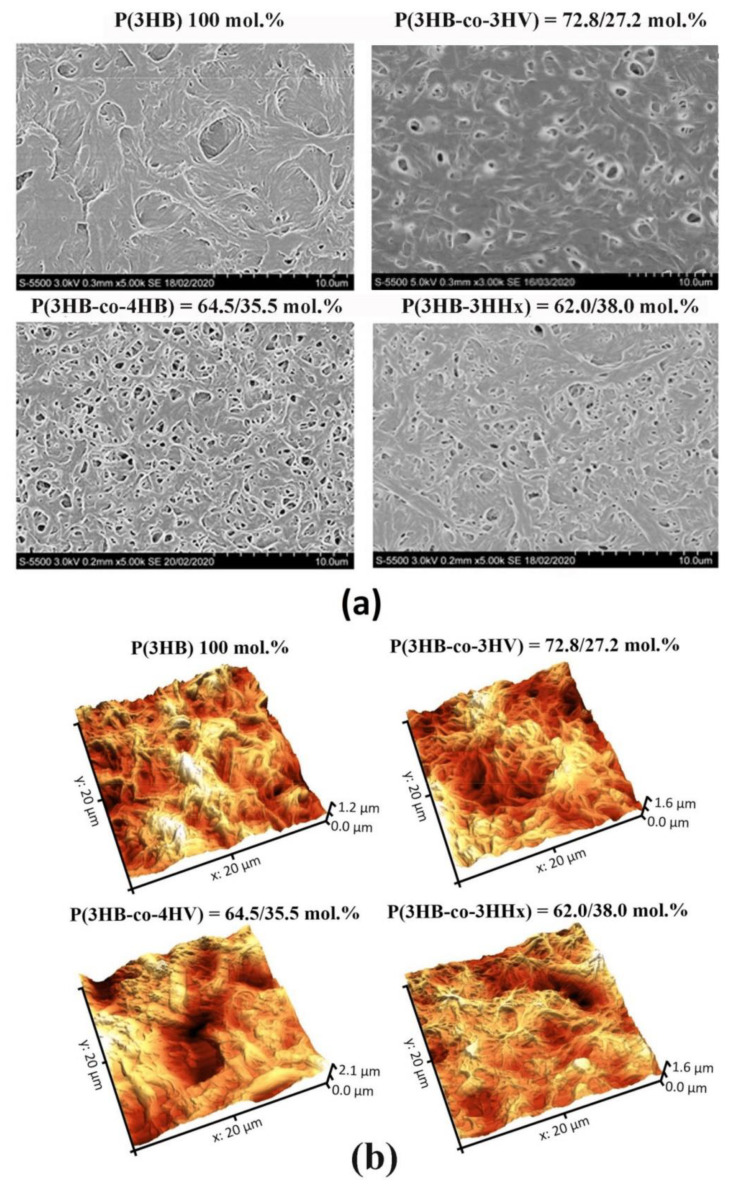
SEM (**a**) and AFM (**b**) images of pristine (non-treated) polymer films prepared from PHAs with different chemical composition.

## References

[1] Volova T.G., Golubev A.I., Nemtsev I.V., Lukyanenko A.V., Dudaev A.E., Shishatskaya E.I. (2021). Laser Processing of Polymer Films Fabricated from PHAs Differing in Their Monomer Composition. Polymers.

